# Relationship of Adiposity and Insulin Resistance Mediated by Inflammation in a Group of Overweight and Obese Chilean Adolescents

**DOI:** 10.1186/1475-2891-10-4

**Published:** 2011-01-14

**Authors:** Marcela Reyes, Sheila Gahagan, Erik Díaz, Estela Blanco, Laura Leiva, Lydia Lera, Raquel Burrows

**Affiliations:** 1 Institute of Nutrition and Food Technology (INTA), University of Chile, Santiago, Chile; 2 Division of Child Development and Community Health, Department of Pediatrics, University of California, San Diego, USA; 3 Center for Human Growth and Development, University of Michigan, Ann Arbor, USA

## Abstract

The mild chronic inflammatory state associated with obesity may be an important link between adiposity and insulin resistance (IR). In a sample of 137 overweight and obese Chilean adolescents, we assessed associations between high-sensitivity C-reactive protein (hs-CRP), IR and adiposity; explored sex differences; and evaluated whether hs-CRP mediated the relationship between adiposity and IR. Positive relationships between hs-CRP, IR and 2 measures of adiposity were found. Hs-CRP was associated with waist circumference (WC) in boys and fat mass index (FMI) in girls. Using path analysis, we found that hs-CRP mediated the relationship between adiposity (WC and FMI) and the homeostatic model assessment of insulin resistance (HOMA-IR) (p < 0.05) in both sexes. Our novel finding is that inflammation statistically mediated the well described link between increased adiposity and IR.

## Introduction

Adolescent obesity is a major public health problem associated with cardiovascular (CV) risk factors including abdominal obesity, insulin resistance (IR), dyslipidemia and hypertension [1,2]. While visceral fat mass is strongly associated with CV risk, causality has not been established [3]. Furthermore, the underlying mechanisms by which excess fat mass (FM) leads to CV risk remain unclear. The mild chronic inflammatory state characterizing obesity may be an important pathophysiologic link between increased adiposity and CV disease [4]. Some investigators have found differences in these associations by sex [5,6]. The purpose of this study was to: assess associations between high-sensitivity CRP (hs-CRP), adiposity and the homeostatic model assessment of insulin resistance (HOMA-IR); explore sex differences in these relationships; and evaluate whether inflammation mediated the relationship between adiposity and HOMA-IR in a sample of overweight and obese Chilean adolescents.

## Methods

The sample was drawn from a larger study of nutrition and physical activity among Chilean adolescents (Tanner stage ≥ 2) attending school in Santiago, Chile (n = 1,780). Overweight and obese adolescents were invited to participate. Exclusion criteria included: chronic diseases; acute infections; hs-CRP values above 9 mg/L; or any medications. Parents signed informed consent and adolescents signed informed assent. Ethics Board of the Institute of Nutrition and Food Technology, University of Chile (INTA) approved this study. A pediatric endocrinologist performed all anthropometric measurements in duplicate. Each adolescent was measured in the Frankfurt position wearing underwear, without shoes. Body mass index (BMI), sex-and age-specific BMI Z-scores, and waist circumference (WC) percentiles were calculated based on the U.S. Centers for Disease Control and Prevention Growth Charts/National Center for Health Statistics standards [7,8]. Arterial blood pressure percentiles were classified according the Updated Second Task Force Report recommendations [9]. FM was evaluated using deuterium isotope dilution according to standard methods [10]. We computed FM index (FMI) as follows: fat mass [kg]/height [m^2^] [11]. After a 12-hr overnight fast, 8 mL of venous blood were collected. The assays (glucose, insulin, hs-CRP, lipids) were performed at INTA.

Statistical analyses were performed using SPSS (version 17.0, SPSS Inc., Chicago, IL, US) and SAS software (version 9.2, SAS Institute, Cary, NC, US). Bivariate associations were determined with Spearman correlation coefficients. We used the Mann-Whitney test to evaluate sex differences in CV risk variables (Table 1). Linear regression analyses were used to determine the relationship between adiposity and log transformed hs-CRP, stratified by sex. Path analysis was performed (EB) using SAS proc CALIS to test whether hs-CRP mediated the relationship between adiposity and IR. In the final path model we adjusted for sex and Tanner stage, however, these variables did not reach statistical significance. Standardized path coefficients and t-values are provided; t-values greater than 1.96 reflect p < 0.05. As the model was fully saturated, with degrees of freedom equal to zero, goodness-of-fit for the overall model could not be estimated. Descriptive fit indices, root mean square error of approximation (RMSEA) and comparative fit index (CFI), were acceptable. Per Hu and Bentler [12], RMSEA close to 0.06 and CFI close to 0.95 are suitable.

**Table 1:** Adiposity and CV Risk, by sex†

	Male	Female
	n = 78	n = 59
Age (year)	14.8 (14.7 - 15.0)	14.7 (14.5 - 14.8)
BMI (z score)	2.1 (2.1 - 2.6)	2.3 (2.1 - 2.5)
WC (cm)	91.3 (90.6 - 94.8)	90.7 (89.5 - 93.9)
FMI (kg/m^2^)*	8.2 (8.1 - 9.2)	11.1 (10.7 - 11.8)
HOMA-IR	2.2 (2.3 - 3.3)	2.1 (1.9 - 2.6)
hs-CRP (mg/L)	0.8 (0.9 - 1.5)	0.7 (0.8 - 1.5)

† Values are median (95% CI)

* Statistically significant difference between males and females (Mann-Whitney test)

## Results

**Figure 1:**
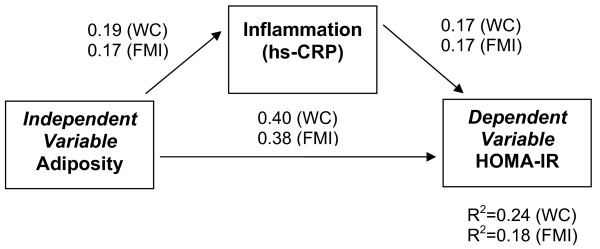
**Pathways between two measures of adiposity--WC (Model 1)^† ^and FMI (Model 2)^‡^--and HOMA-IR: standardized path coefficients**. ^† ^Model 1: WC controlling for sex (β = 0.01) and tanner stage (β = -0.96) Bentler's CFI = 1.00; RMSEA = 0.00. ^‡ ^Model 2: FMI controlling for sex (β = 0.11) and tanner stage (β = -0.41) Bentler's CFI = 0.99; RMSEA = 0.07. All pathways are statistically significant at t > 1.96.

The sample consisted of 78 males and 59 females. Data related to obesity and CV risk factors are shown in Table 1. Adiposity was related to inflammation (log hs-CRP), WC for males (β = 0.36, p < 0.01, R^2 ^= 0.13) and FMI for females (β = 0.37, p < 0.01, R^2 ^= 0.14). Hs-CRP was associated with HOMA-IR in males and females (p < 0.05), independent of adiposity. In our path analysis, hs-CRP partially mediated the relationship between adiposity and HOMA-IR, controlling for sex and Tanner stage (p < 0.05) (Figure 1). For the entire sample, significant paths were found using either measure of adiposity. However, the model using WC as the measure of adiposity explained more of the variance in HOMA-IR than that using FMI.

## Discussion

In our sample of overweight and obese Chilean adolescents, we found that inflammation significantly mediated the relationship between two measures of adiposity, WC and FMI, and increasing levels of HOMA-IR. The association between adiposity and hs-CRP has been extensively studied [13,14]. Likewise, inflammation has been associated with IR [15]. However to our knowledge, there is no other human study showing inflammation as a potential pathophysiologic link between obesity and metabolic derangement, an association well-described in cell-culture and in animal models [16,17].

Gender differences in the relationship between adiposity and inflammation have been previously described in adults with waist-to-hip ratio related to inflammation in men and FM having a stronger association with inflammation among women [6]. Our results make an important contribution, as they indicate that gender differences, described in adults, may also occur in adolescents. During adolescence important sex differences in body composition emerge. Females have more peripheral fat; therefore, WC may not best reflect total fat. On the other hand, males have lower FM, but are likely to have more visceral fat [18]. Measures of adiposity are imperfect and are differentially related to visceral fat. Thus, it is important to choose the measure of adiposity most associated with later adverse outcomes.

Several limitations should be noted. Our findings cannot be generalized to normal weight adolescents or to adolescents from other backgrounds (not Chilean). Future studies should include more direct measures of visceral and subcutaneous fat content (e.g. magnetic resonance or ultrasound imaging), and additional measures of inflammatory status (e.g. interleukin 6, tumor necrosis factor alpha, adiponectin) and insulin sensitivity. It would also be important to assess the magnitude of other factors that might be related to inflammatory status and metabolic performance, for example non-alcoholic steatohepatitis which often occurs with obesity and IR and can progress to inflammation and fibrosis [19,20]. The strengths of the study include careful anthropometric assessment and reliable measurement of FM. Moreover, our subjects were recruited from a community population, rather than using a clinical sample.

In conclusion, our sample of overweight and obese Chilean adolescents showed gender-specific associations between adiposity and systemic inflammation. Additionally, we found that hs-CRP statistically mediated the association between adiposity and IR. This suggests that a systemic inflammatory state is initiated by FM excess even during adolescence and that inflammation influences the metabolic consequences of overweight and obesity. Future research should include 1) detailed assessment of adiposity topography, 2) additional assessments of inflammation (e.g., inflammatory infiltration of adipose tissue) and 3) studies in other populations.

## Abbreviations

BMI: body mass index; CFI: comparative fit index; CV: cardiovascular; FM: fat mass; FMI: fat mass index; HOMA-IR: homeostatic model assessment of insulin resistance; Hs-CRP: high-sensitivity C-reactive protein; INTA: Institute of Nutrition and Food Technology; IR: insulin resistance; RMSEA: root mean square error of approximation; WC: waist circumference.

## Competing interests

The authors declare that they have no competing interests.

## Authors' contributions

MR participated in the design of the manuscript, data analysis, helped to draft the manuscript and significantly reviewed the manuscript before submission. SG participated in the design of the manuscript, data analysis, helped to draft the manuscript and significantly reviewed the manuscript before submission. ED participated in the data acquisition and interpretation and reviewed the manuscript before submission. EB participated in the design of the manuscript, data analysis, helped to draft the manuscript and significantly reviewed the manuscript before submission. LL participated in the data acquisition and analysis and reviewed the manuscript before submission. LL participated in the analysis and reviewed the manuscript before submission. RB participated in the data acquisition, analysis and interpretation, conceived the idea of the manuscript and reviewed the manuscript before submission. All authors read and approved the final manuscript.
